# The impact of substituting general practitioners with nurse practitioners on resource use, production and health-care costs during out-of-hours: a quasi-experimental study

**DOI:** 10.1186/s12875-016-0528-6

**Published:** 2016-09-13

**Authors:** Mieke Van Der Biezen, Eddy Adang, Regi Van Der Burgt, Michel Wensing, Miranda Laurant

**Affiliations:** 1 Radboud Institute for Health Sciences, Scientific Center for Quality of Healthcare, Radboud University Medical Center, P.O. Box 9101, 6500 HB Nijmegen, The Netherlands; 2 Department for Health Evidence, Radboud Institute for Health Sciences, Radboud University Medical Center, P.O. Box 9101, 6500 HB Nijmegen, The Netherlands; 3 Foundation for Development of Quality Care in General Practice, Tilburgseweg-West 100, 5652 NP Eindhoven, The Netherlands; 4 Department of General Practice and Health Services Research, Heidelberg University, INF Marsilius Arkaden, Heidelberg, Germany; 5 Faculty of Health and Social Studies, HAN University of Applied Sciences, P.O. Box 6960, 6503 GL Nijmegen, The Netherlands

**Keywords:** Substitution, Skill mix, General practitioner, Nurse practitioner, Out-of-hours care, Resource use, Costs

## Abstract

**Background:**

The pressure in out-of-hours primary care is high due to an increasing demand for care and rising health-care costs. During the daytime, substituting general practitioners (GPs) with nurse practitioners (NPs) shows positive results to contribute to these challenges. However, there is a lack of knowledge about the impact during out-of-hours. The current study aims to provide an insight into the impact of substitution on resource use, production and direct health-care costs during out-of-hours.

**Methods:**

At a general practitioner cooperative (GPC) in the south-east of the Netherlands, experimental teams with four GPs and one NP were compared with control teams with five GPs. In a secondary analysis, GP care versus NP care was also examined. During a 15-month period all patients visiting the GPC on weekend days were included. The primary outcome was resource use including X-rays, drug prescriptions and referrals to the Emergency Department (ED). We used logistic regression to adjust for potential confounders. Secondary outcomes were production per hour and direct health-care costs using a cost-minimization analysis.

**Results:**

We analysed 6,040 patients in the experimental team (NPs: 987, GPs: 5,053) and 6,052 patients in the control team. There were no significant differences in outcomes between the teams. In the secondary analysis, in the experimental team NP care was associated with fewer drug prescriptions (NPs 37.1 %, GPs 43 %, *p* < .001) and fewer referrals to the ED (NPs 5.1 %, GPs 11.3 %, *p* = .001) than GP care. The mean production per hour was 3.0 consultations for GPs and 2.4 consultations for NPs (*p* < .001). The cost of a consultation with an NP was €3.34 less than a consultation with a GP (*p* = .02).

**Conclusions:**

These results indicated no overall differences between the teams. Nonetheless, a comparison of type of provider showed that NP care resulted in lower resource use and cost savings than GP care.

To find the optimal balance between GPs and NPs in out-of-hours primary care, more research is needed on the impact of increasing the ratio of NPs in a team with GPs on resource use and health-care costs.

**Trial registration:**

ClinicalTrials.gov ID NCT01388374.

## Background

In many Western countries primary healthcare is under pressure due to a rising demand on primary care and rising health-care costs [1–3]. These developments fuel the need for innovative models for organizing health-care delivery more efficiently. Substituting general practitioners (GPs) with nurse practitioners (NPs) is considered worldwide a promising health-care delivery model [4–6]. Substitution of care is feasible since NPs have the ability to treat a large proportion of the complaints presented in primary care autonomously [7–9]. The deployment of NPs has the potential to reduce GPs’ workload, improve efficiency, increase service capacity and improve quality of care [5, 10].

Nurses as GPs’ substitutes in primary daytime practices can provide good quality and safe care, with patient outcomes at least similar to those of GPs [11–14]. Nurse-led care is associated with longer consultation times and lower productivity, an equal number of prescriptions, and equal or more referrals to other services [10, 11, 14]. This would imply that nurse-led care does not necessarily save costs, and might potentially increase costs. Therefore, monitoring the impact of substituting GPs with NPs on resource use and health-care costs is an essential part in the evaluation of skill mix changes [10]. However, only a few studies have investigated the effect of NPs in primary care on health-care costs and the results of the available studies are inconclusive [4, 6, 12, 14]. Outcomes of substitution, resource use and health-care costs in particular are likely to depend on the particular context of care and outcome measures.

Just like in daytime practice, the debate is rising over whether NPs are capable of substituting for GPs in out-of-hours care, where patients present themselves with acute problems. In the Netherlands, GPs provide care for their patients 24/7 and are the gatekeepers to hospital care. As in the UK and Denmark, out-of-hours primary care is most often organized in large-scale general practitioner cooperatives (GPCs). This means GPs take turns in being on duty to take care of all patients within a region outside office hours [15, 16]. Although the deployment of NPs in general practices during daytime is increasing, it is relatively new in the GPCs and there is a lack of evidence about the efficiency of substituting GPs with NPs in those services. Results from daytime are not generalizable to out-of-hours care due to the potentially acute character of the presented symptoms and complaints [17, 18]. As far as we know, there hasn’t been a study conducted on the impact of nurses substituting in out-of-hours primary care on resource use and health-care costs.

## Methods

### Aim

To evaluate the effect of substituting GPs with NPs in out-of-hours care on resource use, production and health-care costs.

### Design

Pragmatic quasi-experimental trial comparing two types of teams providing out-of-hours primary care. In the experimental arm, care is provided by a team of four GPs and one NP, from 10 a.m. – 5 p.m. on a weekend day. In the control arm, care is provided by a team of five GPs on the other weekend day from 10 a.m. – 5 p.m. In addition, care provided by the NPs is compared to that of GPs in the experimental arm.

### Study setting

The evaluation was part of a quasi-experimental study, which was conducted at a general practitioner cooperative (GPC) situated within a hospital next to the Emergency Department (ED) in the south-east of the Netherlands. In this GPC, GPs work in shifts from 5 p.m. – 8 a.m. on weekdays and the entire weekend to take care of a population of approximately 304,000 people. All patients in need of acute care during out-of-hours contact the GPC via a single, regional telephone number where triage nurses decide whether patients receive telephonic advice, a consultation at the GPC, a home visit or referral to the ED. Patients who receive a consultation at the GPC are scheduled in a common presentation list. GPs and NPs choose attending patients from this presentation list [16].

### Study population

#### General practitioners and nurse practitioners

A sample of five NPs and 138 GPs participated in this study. GPs’ mean age was 49.3 years (SD 9); 60 % were male and on average the GPs had been associated with the GPC for 7.3 years (SD 3.7).

All NPs had at least five years of experience working as a licensed NP in primary care or elderly care. None of the NPs had experience working at the GPC prior to the study. Therefore, they received three half days of additional training in commonly presented complaints during out-of-hours [16]. In the Netherlands, the title ‘Nurse Practitioner’ is protected by law and exclusively reserved for those who have completed a Master Advanced Nursing Practice (NLQF/EQF level 7; accredited by the NVAO), and are registered in the specialist register. All NPs have previous experience in nursing at Bachelor of Nursing level. NPs have the authority to independently indicate and perform reserved procedures (including prescribing medicines) in his/her area of expertise using the same guidelines as GPs [19, 20]. This is a major difference from the widely implemented practice in the Netherlands whereby practice nurses take care of patients with chronic complaints following evidence based protocols. These practice nurses are usually operating at a Bachelor of Nursing level (NLQF/EQ Level 6) and are, in contrast to NPs, always working under supervision of a GP and not authorised to diagnose and prescribe medicine autonomously [21].

Based on the educational training of the NPs, the GPC in this study excluded the following patients from NP care: those younger than one year and those with psychiatric complaints, abdominal pain, chest pain, a neck ailment, headache or dizziness. Based on the information of the triage nurse, NPs decided which patients from the common presentation list they would call in for consultation. Patients excluded from NP care would receive consultation from a GP. In cases where the complaint of the patient during the triage was different from the complaint during the consultation, NPs were allowed to decide autonomously whether they felt competent or not to complete the consultation themselves, whether they consulted the GP about the patient or whether to refer the patient to the GP.

#### Patients

All patients who visited the GPC during the data collection were included in the study. Due to the explorative character of the study a statistical power calculation could not be done reliably. In order to get reasonably accurate estimates, a 15-month follow-up was chosen to get a sufficiently large sample.

### Randomization

The experimental and control days were rotated systematically between Saturday and Sunday. The five-week rotation scheme was determined in advance. Days were randomized between Saturday and Sunday to avoid bias due to possible differences in patient presentations on those weekend days. Patients were unaware of experimental or control days when they contacted the GPC. The GPs were randomly assigned to the weekend days; they did not know whether they would work with an NP at the time of scheduling.

### Measures and data collection

The primary outcome was resource use following a consultation at the GPC. Resource use included X-rays, drug prescriptions and referrals to the ED. Other imaging tests or laboratory samples than X-rays could not be ordered by the providers. If such diagnostic tests were necessary patients were referred to the ED or to their own GP the next day. Data related to resource use were measured as dichotomous outcome variables.

Secondary outcomes were production per hour (indicated as the mean number of patients per care provider per hour) and direct health-care costs. Direct health-care costs were based on personnel costs (based on production per hour and salary) and costs per unit of resources used for each consultation (X-rays, drug prescriptions and referrals to the ED). Here volumes are combined by unit prices that constitute costs.

Data abstracted to compare baseline characteristics included potential confounders for the comparison: age (in four categories), urgency (in five categories), gender, and type of complaint (indicated as an International Classification Primary Care [ICPC] code). All data were abstracted from the electronic medical patient records at the GPC and coded by the care providers as part of their routine during the consultation.

Data were collected from April 2011 to July 2012.

### Analysis

#### Baseline characteristics

Baseline characteristics of the study population are presented as a proportion (%) since all measures (age, gender, urgency level and type of complaint (ICPC)) were measured in categorical variables. Differences between the experimental arm and control arm were tested using a Chi^2^ test. The same analysis was performed in secondary analysis comparing baseline characteristics between patients treated by the NP and patients treated by the GP in the experimental arm.

#### Resource use

Resource use (i.e., X-rays, drug prescriptions and referrals to the ED) was evaluated by analysing differences in volumes between groups. Logistic regression analysis for dichotomous outcomes was conducted to compare the two study arms. To adjust for potential confounders a second logistic regression model was used that corrected for age, gender, urgency level and ICPC group. The same analysis was performed in the secondary analysis to compare the NPs and GPs in the experimental arm.

#### Production per hour

Production per hour was calculated by dividing the total number of patients per care provider by the exact number of hours per care provider. This resulted in a mean number of patients treated per hour per care provider. A linear mixed model was used to test the differences in production per hour between the teams. Results were corrected for holidays, weekend days, number of professionals and the total number of patients per day. The same analysis was performed in the secondary analysis to compare the NPs and GPs in the experimental arm.

#### Direct health-care costs

The economic evaluation was designed as a cost-minimization analysis, considering direct health-care costs of the consultation only. In this analysis, based on previous study reviews, patient outcomes of the two study conditions are assumed to be equal [22]. Direct costs were calculated for each consultation separately including costs for care provider, X-rays, drug prescriptions and referral to the ED.

Costs for the GP and NP time per consultation were calculated by dividing the tariff per hour by the mean production per hour. For NPs the tariff was based on their salary from the GPC, including social security contributions (approximately 40 %) and premium pay (50 %). For GPs the tariff was based on the payment agreements with health insurance companies. This tariff is calculated on the basis of a total tariff per GPs’ patients for providing 24/7 care.

The tariff valid for the GPC per care provider per hour was €77 for GPs, and €65,46 and €66,38 for NPs (see Table 1). Next, following the guidelines of the Dutch Manual for Costing, the cost for each referral to the ED was set at €151 and €43,98 and €45,37 for an X-ray [23]. As a result of the differences between the minimum and maximum price for medicine, two separate costs were calculated per drug prescription. All the direct health-care costs were calculated using the tariffs that were valid for the intervention period (see Table 1).

**Table 1:** Prices per unit in 2011-2012

Resource	Unit	Costs (€)	Data source
Salary costs GP	Hour	€77	GPC (based on agreements with health insurance companies)
Salary costs NP	Hour	€65,46 (as per 1-4-2011)	GPC
		€66,38 (as per 1-4-2012)	
Drug prescription	Consultation	Variable (minimum and maximum prices)	http://www.medicijnkosten.nl/ (indicated by Dutch Manual for Costing [23])
X-ray	Consultation	2011: €43,98	The Dutch Healthcare Authority (NZa) (indicated by Dutch Manual for Costing [23])
		2012: €45,37	
Referral to the Emergency Department	Consultation	€151	Dutch Manual for Costing [23]

To provide further insight into the cost differences, a *t*-test was performed to compare the unadjusted estimates between the experimental and control arm. Second, to adjust for potential confounders a linear regression model was used that corrected for case mix (i.e., age, gender, urgency level, ICPC group). For the cost of drug prescriptions the minimum price per medicine was used in the primary analysis. Deterministic uncertainty was explored by one-way sensitivity on costs of drug prescriptions by including the maximum price per medicine. The same analysis was used in the secondary analysis to compare NPs and GPs in the experimental arm.

Finally, we applied a bootstrapping procedure (with 1,000 replications) to manage the highly skewed costs across patients. The statistical analysis, including the bootstrapping, was carried out using SPSS software version 22 (SPSS Inc, Chicago, IL, USA).

## Results

**Fig.1:**
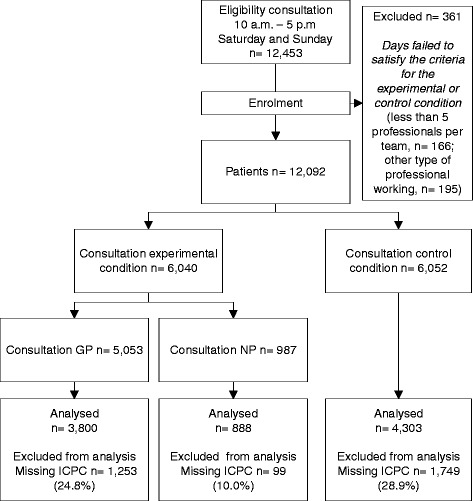
Flow diagram of the study

The experimental arm included 34 Saturdays and 29 Sundays (63 intervention days), and the control arm included 29 Saturdays and 34 Sundays (63 control days). In total, 12,092 patients had a consultation during the study period. In the experimental arm, 987 patients visited an NP and 5,053 patients visited one of four GPs. In the control arm, 6,052 patients visited one of five GPs. A total of 3,101 cases (10.0 % with an NP, 27.0 % with a GP) could not be analysed due to a missing ICPC code (a flow diagram of the study is shown in Fig. 1).

### Baseline characteristics

There were no significant differences in patient characteristics between the experimental and the control arm (Table 2 shows the 10 most presented complaints). However, as expected given the exclusion criteria, significant differences were found between GPs and NPs for patients’ age (*p =* .002), urgency level (*p <* .001) and type of complaint (*p <* .001) [18]. GPs saw more patients aged >64 years, with an urgency level of U2, and suffering digestive, cardiovascular and neurological complaints. NPs saw more patients suffering skin and respiratory complaints and with an urgency level of U4.

**Table 2:** Baseline characteristics, top 10 ICPC groups

	Control arm	Experimental arm	GP Experimental arm	NP Experimental arm
Complaints (%)^a^				
Skin	21.7	22.7	20.7	31.2
Musculoskeletal	20.5	20.1	19.6	22.2
Respiratory	15.2	14.2	13.7	16.3
Digestive	10.5	9.9	11.4	3.0
Eye	6.0	6.1	6.5	4.4
General and unspecified	5.9	6.5	6.6	6.0
Ear	5.7	5.8	5.6	6.8
Urological	5.5	5.7	5.7	5.6
Cardiovascular	2.5	2.5	2.9	0.7
Neurological	2.3	2.3	2.8	0.3
Other	4.2	4.2	4.5	3.5

Tested using a Chi^2^ test

^a^significant difference between the GP and NP in the experimental arm

### Resource use

#### Experimental arm vs control arm

Table 3 shows both the unadjusted and adjusted differences in X-rays, drug prescriptions and referrals to the ED. Across the overall sample, the team in the experimental arm compared to the control arm less often ordered an X-ray (4.4 % vs. 5.3 %; *p =* .017), less often prescribed drugs (42.0 % vs. 44.1 %; *p =* .022) and less often referred patients to the ED (10.2 % vs. 11.6 %; *p =* .02). However, none of these differences remained significant after adjusting for case mix (i.e., age, gender, urgency level, ICPC group).

**Table 3:** Rate differences of resource use following a visit to the GPC

	Experimental vs control arm				Experimental arm GP vs NP			
		95 % CI for exp *b*				95 % CI for exp *b*		
	B (SE)	Lower	Exp *b*	Upper	B (SE)	Lower	Exp *b*	Upper
Unadjusted estimates								
X-ray	-.202* (.09)	.692	.817	.965	-.303 (.156)	.544	.738	1.002
Drug prescription	-.084* (.037)	.855	.919	.988	.246 *** (.072)	1.111	1.279	1.472
Referral ED	-.136* (.058)	.779	.873	.979	.866 *** (.152)	1.766	2.378	3.202
Adjusted estimates								
X-ray	-.203 (.11)	.682	.816	1.006	-.168 (.19)	.588	.846	1.115
Drug prescription	-.09 (.05)	.838	.916	1.001	.317 *** (.077)	1.167	1.373	1.616
Referral ED	-.13 (.07)	.759	.877	1.014	.60** (.179)	1.277	1.814	2.576

Tested within a logistic regression model. Adjusted estimates are adjusted for age, gender, urgency level and ICPC group

* *p* < .05

** *p* < .01

*** *p* < .001

#### NPs vs GPs in the experimental arm

NP care was associated with fewer drug prescriptions (37.1 % vs. 43 %; *p <* .001) and fewer referrals to the ED (5.1 % vs 11.3 %; *p < .*001) than GP care. These differences remained significant after adjusting for case mix. There was no statistical significant difference between NPs and GPs with regard to ordering X-rays (NPs 5.6 % vs. GPs 4.2 %).

### Production per hour

The mean production per professional was 2.9 consultations per hour in both the experimental arm and the control arm. In the experimental arm the mean number of consultations per hour was 3.0 for GPs and 2.3 for NPs (*p* < .001).

### Direct health-care costs

Based on the tariff per hour and the production per hour, the mean costs per GP consultation were calculated at €25,67 and the costs per NP consultation were calculated at €27,28 (as per April 2011) and €27,66 (as per April 2012).

#### Experimental arm vs control arm

Table 4 presents the unadjusted and adjusted cost differences between the experimental and the control arm. The mean costs of a consultation in the experimental arm were €2,05 less than a consultation in the control arm (95 % CI: €-3,79; €-0,29; *p =* .02). However, this difference did not remain significant after correcting for case mix (i.e., age, gender, urgency level, ICPC group). In the sensitivity analysis with the maximum cost per medication the adjusted difference remained non-significant (95 % CI: €-3,65; €0,15).

**Table 4:** Unadjusted and adjusted differences in direct health-care costs following a consultation at the GPC

	Experimental vs control arm			
	experimental arm	control arm	Mean difference	95 % CI
Unadjusted mean cost per consultation (minimal medication costs)	€44,93	€46,98	€-2,04*	€-3,79; €-0,29
Adjusted mean cost per consultation (minimal medication costs)			€-1,53	€-3,36; €0,46
	Experimental arm GP vs NP			
	GP	NP	Mean difference	95 % CI
Unadjusted mean cost per consultation (minimal medication costs)	€46,17	€38,59	€-7,58**	€-10,82; €-4,34
Adjusted mean cost per consultation (minimal medication costs)			€-3,34*	€-5,97; €-0,65

Tested within a linear regression model. Adjusted estimates are adjusted for age, gender, urgency level and ICPC group

* *p* < .05

** *p* < .001

#### GPs and NPs in the experimental arm

The mean cost per consultation on the experimental day was €7,58 less for a consultation with an NP than for a consultation with a GP (95 % CI: €-10,82; €-4,34; *p* < .001) (see Table 4). After correction for case mix a significant difference of €-3,34 remained in favour of the NP (95 % CI: €-5,97; €-0,65; *p =* .02). The main influence on the difference in costs was the number of patients referred to the ED. In the sensitivity analysis with the maximum costs per medication the adjusted difference between the experimental and control arm increased to €-3,51 (95 % CI: €-6,77; € -0,24; *p =* .04).

## Discussion

### Statement of principal findings

This study did not find a significant difference between teams with an NP and teams with only GPs with regard to X-rays, drug prescriptions and referrals to the ED. Moreover, the production per hour and the cost per consultation for the team with an NP were not different from teams with only GPs.

In the experimental team, NP care was found to be associated with significantly fewer drug prescriptions and fewer ED referrals than care delivered by GPs. NPs were shown to have a lower production per hour than GPs. The cost per consultation with an NP was lower than with a GP.

### Strengths and weaknesses

A strength of the current study is its large patient sample and a long follow-up period, but limitations include the single-centre character of the study and the low number of nurse practitioners involved. Moreover, we had a relatively large number of missing ICPC codes. There appeared to be only a few GPs who repeatedly did not report ICPC codes, which means the bias is related to the GP and not the ICPC diagnosis or day. This is supported by the fact that the ICPC codes in our study are comparable to those of other out-of-hours services in Western countries [18]. Therefore, we don’t suspect that the missing ICPC codes will cause any bias to our outcomes.

It should be noted that the current study shows the effect of NPs within a GPC. Although many countries have organized out-of-hours care in large-scale organizations in previous years, the various types of health-care systems influence the generalizability of the research findings [15]. Moreover, the education and deployment of NPs differs between, and even within, countries and health-care systems. In the Netherlands, as in most countries, NPs providing care are always working as part of primary care teams alongside GPs [21, 24]. Our results can therefore not be generalized to other models of care in which NPs are working in teams without GPs [25]. Moreover, in the current study the NPs were primarily responsible for treating minor ailments. The complexity of tasks can differ between regions and countries.

In the current study, NPs with no experience working at the GPC at the start of the study were compared with GPs who had on average 7.3 years of experience at the GPC. This may have influenced resource use or production per hour. A strength of the current study is the fact that researchers did not change patient allocation, which gives an accurate representation of the daily practice and related cost estimates.

We only included costs relevant from the GPCs’ viewpoint (tariff per hour, production per hour) and direct health-care costs relevant from health insurance companies’ viewpoint (X-rays, drug prescriptions and referrals to the ED). This implies that it is not possible to draw conclusions on whether the deployment of NPs is cost saving from a societal viewpoint. Therefore, other factors, such as the difference in costs of training, rates of sick leave, patient follow-up after a GPC visit or after ED referral, et cetera, should have been included [23, 26].

### Comparisons with other studies

Meta-analyses based on research conducted in daytime primary care did not show differences between nurses and GPs in terms of prescriptions, diagnostic test orders and referrals [10]. Although, in line with these meta-analyses, we did not find differences at team level, our secondary analysis in the experimental team showed a difference between GPs and NPs in terms of drug prescriptions and referrals to the ED. We cannot determine whether this difference in resource use is an overuse of medication or referrals by GPs, or an underuse by NPs. There is no capacity to examine how clinical outcomes would differ from the likely outcomes if patient care was provided by the other care provider [27]. Inappropriate referrals and prescriptions may further increase health-care costs and unnecessary treatments in the hospital. Based on reviews of research, we do not expect an underuse by NPs since patient outcomes in primary care were found to be at least equivalent for NPs and GPs [12, 14]. Moreover, research on the ED and hospital care shows that the diagnostic accuracy of NPs is comparable to that of doctors [28, 29].

We found a lower production per hour for NPs than for GPs. However, it was not possible to adjust this outcome for case mix. This makes comparison between GPs and NPs difficult since they treat different patients. However, we expect the number of consultations per hour to be a reliable measure. This is supported by the fact that our outcomes are comparable to results from meta-analyses on consultation times [10]. Besides treating different patients, lower production per hour can also be associated with less experience [30]. Although NPs had at least five years of experience in primary or elderly care, none of them had any experience in out-of-hours primary care at the start of the study. Other possible explanations for longer consultations include a higher use of protocols [10], and a more holistic approach and greater provision of information by NPs than by GPs [31]. In addition, the provision of more health education and information by NPs may result in fewer prescriptions [32].

Based on previous research, we expected NP care to be cost saving due to a lower salary for NPs than for GPs [33]. However, in line with another study, lower production per hour appeared to lessen the influence of salary differences on consultation costs [34]. Another reason for the small influence of salary costs on overall costs is the small difference in tariff between the GPs and NPs during out-of-hours care. This is because the GPs receive financial compensation for out-of-hours care based on the total tariff for providing care to their patients 24/7. This means that the GPs receive a fixed tariff, whereas the tariff per hour for NPs was based on their gross salary including social security contributions and premium pay. The differences in tariff per hour would have been bigger in cases where the care providers were employed by the GPC in the same way. For example, the difference in gross salary of a GP employed by another GP and the NPs in our study is approximately 60 % [35]. In another Dutch study in daytime primary care, the salary of an NP appeared to be less than half of that of a GP. As a consequence, in that study, cost differences were mainly caused by the difference in salary [36]. It is expected that bigger differences in salary will result in more cost savings when GPs are substituted with NPs.

The current study shows that the differences in referral rates to the ED strongly influenced consultation costs. The fewer referrals by NPs resulted therefore in lower mean costs of care provided by NPs than by GPs. It is difficult to compare these findings with previous research due to conflicting results on the effect of substituting GPs with NPs in primary care on the cost of health care. Moreover, due to heterogeneous outcome reporting and the small number of studies they are hard to interpret. However, in general, NP care seems to be associated with lower or equal health-care costs per consultation [6, 12]. Only one study found increased costs associated with NP care. These results were based on two factors that we did not measure: time spent by GPs on supervising and number of return visits [34]. The time spent on supervising in the current study was, however, relatively low. The NPs consulted a GP in only 7.1 % of all consultations. Only 0.2 % of the patients were taken over by the GP; the other consultations were completed by the NP. Consultations between the NP and GP are considered part of daily practice and comparable to consultations GPs have with other GPs Therefore, we do not expect this to bias our outcomes.

### Study implications

The current study shows no differences in resource use and direct health-care costs between teams with an NP and teams with GPs only. Therefore we conclude that during out-of-hours, involvement of NPs in multidisciplinary teams can increase capacity without increasing resource utilization.

Our results show that using NPs as substitutes for GPs in out-of-hours care is a feasible solution for decreasing GPs’ workload or increasing service capacity. It should be noted that tasks at GPCs are limited to providing acute care and do not use NPs’ competences to the full. Tasks such as preventive projects, psycho‐social home visits, providing ongoing training for staff and developing protocols are only performed during the daytime. In countries where GPs deliver 24/7 care, the implementation of NPs in primary care will only succeed when they (just like GPs) provide care 24/7.

With the need for extra workforce in primary care, our data suggests that substitution by NPs can be considered an solution economical equal to the care delivered by GPs. However, because we only included one GPC, and only measured direct costs, results should be interpreted with caution. Economic evidence on which to make judgments on future out-of-hours care is far more complicated [37]. Other costs from a societal perspective such as training cost and unemployment rates of physicians in hospital care have to be taken into account. This implies that decisions on the substitution of GPs by NPs in out-of-hours primary care should not only depend on costs, but on other factors such as a view on professional roles, responsibilities, and quality and safety of care [34].

As this study showed a significant difference in cost per consultation in favour of NPs, it may be possible that deploying more NPs in a team with GPs is more cost saving. Future research is needed to indicate an optimal balance in which teams with NPs and GPs provide the most efficient care for patients in out-of-hours primary care.

## Conclusion

The current study indicated no differences between teams with an NP and teams with only GPs with regard to resource use, production per hour and direct health-care costs. However, in teams with an NP, the NP appeared to make fewer drug prescriptions and fewer referrals to the ED than the GPs. Due to lower resource use, the cost of a consultation with an NP was less than that of a consultation with a GP. The current study shows that involvement of NPs in teams with GPs can increase capacity without increasing resource utilization during out-of-hours. More research is needed to find the optimal balance between GPs and NPs to cover all patient care in out-of-hours primary care efficiently. Obviously, decisions on substituting GPs with NPs should be based on the full range of considerations, including a view on the professional roles and responsibilities of NPs in of out-of-hours care, rather than just arguments related to resource use and costs.

## Abbreviations

ED: Emergency Department
GP: General practitioner
GPC: General practitioner cooperative
ICPC: International Classification Primary Care
NP: Nurse practitioner
